# Metataxonomic and metaproteomic profiling of the oral microbiome in oral lichen planus - a pilot study

**DOI:** 10.1080/20002297.2022.2161726

**Published:** 2022-12-28

**Authors:** Maria Bankvall, Miguel Carda-Diéguez, Alex Mira, Anders Karlsson, Bengt Hasséus, Roger Karlsson, Jairo Robledo-Sierra

**Affiliations:** a Department of Dental Medicine, Karolinska Institute, Huddinge, Sweden; b Department of Health and Genomics, Center for Advanced Research in Public Health, FISABIO Foundation, Valencia, Spain; c School of Health and Welfare, Jönköping University, Jönköping, Sweden; d Nanoxis Consulting AB, Gothenburg, Sweden; e Department of Oral Medicine and Pathology, Institute of Odontology, The Sahlgrenska Academy, University of Gothenburg, Gothenburg, Sweden; f Clinical microbiology, Sahlgrenska University Hospital, Gothenburg, Sweden; g Faculty of Dentistry, CES University, Medellin, Colombia

**Keywords:** Oral cavity, oral mucosa, oral lichen planus, oral microbiome, oral bacteria, metataxonomics, metaproteomics

## Abstract

**Background:**

A growing body of evidence demonstrates a different bacterial composition in the oral cavity of patients with oral lichen planus (OLP).

**Patients and methods:**

Buccal swab samples were collected from affected and non-affected sites of six patients with reticular OLP and the healthy oral mucosa of six control subjects. 16S rRNA gene MiSeq sequencing and mass spectrometry-based proteomics were utilised to identify the metataxonomic and metaproteomic profiles of the oral microbiome in both groups.

**Results:**

From the metataxonomic analysis, the most abundant species in the three subgroups were Streptococcus oralis and Pseudomonas aeruginosa, accounting for up to 70% of the total population. Principal Coordinates Analysis showed differential clustering of samples from the healthy and OLP groups. ANCOM-BC compositional analysis revealed multiple species (including P. aeruginosa and several species of Veillonella, Prevotella, Streptococcus and Neisseria) significantly over-represented in the control group and several (including Granulicatella elegans, Gemella haemolysans and G. parahaemolysans) in patients with OLP. The metaproteomic data were generally congruent and revealed that several Gemella haemolysans-belonging peptidases and other proteins with inflammatory and virulence potential were present in OLP lesions.

**Conclusion:**

Our data suggest that several bacterial species are associated with OLP. Future studies with larger cohorts should be conducted to determine their role in the aetiology of OLP and evaluate their potential as disease biomarkers.

## Introduction

Oral lichen planus (OLP) is one of the most common oral mucosal diseases, with a global prevalence of 0.49–1.43% [1,2]. The lesions are usually characterised by bilateral whitish striae forming a reticular pattern and erythema, but papules, plaques, and ulcerative features may arise. For the proportion of affected individuals with symptoms – around 40%– [3], this condition is exceptionally debilitating as the pain in the oral mucosa may be intense and widespread. Currently, there is no cure, but there are symptom-relieving preparations available. Patients with OLP also carry an increased risk of developing oral cancer of about 1.1% [4,5].

Despite a tremendous scientific effort to understand the aetiology behind OLP over the past two decades, the exact mechanism is yet to be elucidated. Previous studies have shown that T cells are involved in its pathogenesis, but their role in this specific process is unclear [6]. Presumably, a still unknown exogenous antigen, autoantigen, or superantigen elicits an inflammatory response in the oral epithelium leading to apoptosis of basal epithelial keratinocytes, which manifests as chronic inflammation [7]. OLP likely represents several different variants of oral lichenoid lesions. Therefore, the condition should be regarded as a reaction pattern with shared histopathological characteristics rather than a distinct disease in the traditional sense. More plausible is the theory that multiple antigens, combined with various environmental factors, trigger an inflammatory response in genetically predisposed individuals where the antigen may vary among subjects. Thus, the aetiology is expected to be multifactorial, as in most other disease states, and not monocausal, which is the approach most researchers have used to study OLP.

Perturbations to the structure of complex commensal microbiota communities in, for example, the gut and oral cavity modulate innate and adaptive immune responses resulting in immune-mediated conditions such as inflammatory bowel disease and periodontitis, where an increase in the number or activity of pathogenic microbiota has been shown to cause microbial dysbiosis and immune alterations, or induce inflammatory pathways [8,9]. Studies specific to OLP have demonstrated that intensive oral hygiene and plaque control may improve gingival lichen planus lesions [10,11] and that treatment with chlorhexidine and antibiotics can regress the lesions and relieve symptoms considerably [12,13], suggesting a role for the oral microbiota in the onset or exacerbation of OLP. The involvement of the oral microbiome has been addressed in other inflammatory oral mucosal disorders such as recurrent aphthous stomatitis [14,15] and geographic tongue [16], as well as in other autoimmune diseases such as Sjögren’s syndrome [17], rheumatoid arthritis [18], and systemic lupus erythematosus [19]. Interestingly, recent studies on neurological and chronic pain disorders such as burning mouth syndrome also show involvement of the oral microbiota [20]. Although the exact role of the oral microbiome in the pathogenesis of these conditions remains elusive, it is realistic to assume that it could also be implicated in OLP.

The role of viral infections in the development of OLP has been studied extensively in the hepatitis C virus [21,22] and human papillomavirus [23,24], but studies on the association between oral bacteria and OLP are just emerging. There seems to be a shift in the mucosal bacterial composition and salivary microbiota in patients with OLP compared to healthy control subjects, as well as a difference between affected and non-affected sites in the same patient [25–29]. Additionally, certain species have been reported to damage the epithelial barrier, invade the lamina propria, and internalise epithelial cells or T cells, resulting in the production of specific T-cell chemokines [26]. Therefore, bacterial dysbiosis has been proposed to be a causative factor of periods of exacerbation in OLP and the clinical changes that occur to the lesions over time [28].

Our overall hypothesis is that in a particular group of patients with OLP, the lesions arise as an inflammatory response to one/several antigens from one/several oral bacterial species. By exploring the metataxonomic and metaproteomic profiles of the oral microbiome of affected and non-affected sites in patients with OLP compared to healthy controls, we hope to provide new insights into the aetiology of this condition, as adequate treatment regimens with a possible curative potential still lack today.

## Materials and methods

### Study population

Patients with OLP who attended regular follow-up appointments at the Clinic of Oral Medicine, Public Dental Health, Gothenburg, Sweden, were invited to participate in the study. Oral medicine specialists established the diagnosis of OLP following the clinical and histopathological criteria set by the World Health Organization [30]. We included only patients with bilateral reticular lesions, as we expected erythematous and ulcerative lesions to have a microbial composition unrelated to the disease onset or exacerbation. Moreover, they had not received local or systemic treatment for OLP for at least six months before sampling. Patients who presented with different types of oral mucosal lesions, including lichenoid contact reactions observed at the lateral border of the tongue or on the buccal mucosa and adjacent to amalgam restorations, were excluded from the study. Further exclusion criteria were the use of antibiotics or antibacterial mouth rinses in the previous six months, smoking or excessive alcohol consumption (i.e. more than three times weekly). After applying the inclusion and exclusion criteria, we enrolled six patients with OLP (females, n = 4; mean age, 57.6 years; age range, 38–71). As a control group, we included six age- and gender-matched volunteers (females, n = 4; mean age, 57.1 years; age range, 38–66) with no oral mucosal lesions.

### Sampling procedures

Three samples were taken from patients with OLP: two from OLP lesions located at each side of the buccal mucosa (OLP) and one from non-affected oral mucosa (buccal sulcus or labial commissure) (H-OLP). Two samples were taken from the control subjects (H): one from each side of the healthy buccal mucosa. The participants were prevented from brushing their teeth, drinking, and eating for at least one hour before sampling. The same oral medicine specialist (JR-S) collected all the samples with Copan ESwab^TM^ 480C (Copan Diagnostics Inc., Murrieta, CA, USA) using a standardised technique according to the manufacturer’s protocol. In brief, the sterile swab was used to obtain bacteria by stroking with gentle downward pressure ten times in one direction, turning the swab 180°, and stroking ten times in the same manner. Each swab was placed in the sterile Liquid Amies Medium (1 ml) provided by the manufacturer and stored at 4°C immediately after the sampling procedure. Within 2 hours, the samples were homogenised with a vortex for 10 seconds and transferred to two lysis tubes containing skimmed milk, tryptone, glucose, and glycerine (STGG) medium (500 µl to each tube) and stored at −20°C until subsequent processing. The samples were coded, rendering the biochemical analysts blind to group status.

### DNA extraction and sequencing

Samples were extracted using the MagNa Pure LC DNA Isolation kit II and a MagNa Pure Instrument (Roche Molecular Systems Inc., Pleasanton, CA, USA). The protocol was used as indicated by the manufacturer with some modifications following Dzidic et al [31]. In brief, samples were lysed using 3 × 10-second cycles of ultrasound and enzymatic digestion with an enzyme cocktail of lysozyme (100 mg/ml), lysostaphin (5 kU/ml), and mutanolysin (2.5 kU/ml). Finally, proteins were degraded using Proteinase K. Then, the DNA was resuspended in 100 μl of ultrapure DNAse-free water.

After measuring the DNA by fluorimetry, the V3-V4 hypervariable region of the 16S rRNA gene was amplified using universal primers optimised for Illumina sequencing, following Dzidic et al [31]. The library was constructed using the Metagenomic Sequencing Library Preparation Illumina protocol (Part #15044223, Rev. A) and sequenced with the standard procedure recommended by the manufacturer at the sequencing service in FISABIO (Valencia, Spain) using 2 × 300 bp paired-end sequencing with an Illumina MiSeq instrument. Data has been deposited in the SRA database (Bioproject: PRJNA598825).

### 16S rRNA gene data analysis

Reads were analysed as previously described by Hernandez et al [32], using DADA2 [33]. Briefly, paired reads were filtered, end-trimmed, denoised, and merged before adapters and primers were filtered out. Singletons and PCR chimeras were removed. The remaining reads were assigned to a taxon using the SILVA non-redundant database [34]. Reads were compared by blast, and those ≥97% identical were considered amplicon sequence variants (ASVs). Rarefaction curves and diversity analyses were performed using the same number of reads for all samples. ANCOM-BC approach was used to normalize and compare the abundance of taxons to account for the compositional nature of 16S ribosomal rRNA sequencing data [35].

### Protein digestion

Samples collected were thawed and the MolYsis™ kit (MolYsis Basic5 kit, Molzym GmbH & Co., Bremen, Germany) was used to remove human biomass, according to the supplier’s protocol, with minor modifications [36]. The resulting bacterial pellets were resuspended in 150 µl ammonium bicarbonate (20 mM) buffer containing 1% sodium deoxycholate (SDC) final concentration (from 5% stock solution in 20 mM ammonium bicarbonate). Bacterial cells were lysed by bead beating, and the supernatants were collected. Trypsin (2 µg/ml, 100 µl ammonium bicarbonate, 20 mM pH 8) was added, and samples were digested for approximately 8 h at 37°C. Sodium deoxycholate was removed by acidification with 40 µl 10% FA (formic acid), samples were centrifuged at 10,000×g for 5 minutes, and the supernatants were transferred to new tubes. The supernatants containing peptides were then desalted (Pierce™ Peptide Desalting Spin Columns, Thermo Fisher Scientific, Waltham, MA, USA) according to the manufacturer’s instructions.

### Proteomic analysis and protein identification

The peptide samples and fractions were dried, reconstituted in 3% acetonitrile and 0.2% formic acid, and analysed on a QExactive HF mass spectrometer interfaced with the Easy-nLC1200 liquid chromatography system (Thermo Fisher Scientific). Peptides were trapped on an Acclaim Pepmap 100 C18 trap column (100 μm x 2 cm, particle size 5 μm, Thermo Fischer Scientific) and separated on an in-house packed analytical column (75 μm x 35 cm, particle size 3 μm, Reprosil-Pur C18, Dr Maisch), using a gradient from 5% to 80% acetonitrile in 0.2% formic acid over 90 min at a flow of 300 nL/min. The instrument operated in a data-dependent mode where the precursor ion mass spectra were acquired at a resolution of 60,000, m/z range 400–1600. The ten most intense ions with charge states 2 to 4 were selected for fragmentation using HCD at a collision energy setting of 28. The isolation window was set to 1.2 Da and dynamic exclusion to 20 s and 10 ppm. MS2 spectra were recorded at a resolution of 30,000 with a maximum injection time set to 110 ms.

Identification was performed using Proteome Discoverer version 2.4 (Thermo Fisher Scientific). The database search was performed using the Mascot search engine v. 2.5.1 (Matrix Science, London, UK), matching with the ‘3042 microbiome+Gemella’ ‘3042_combined_210322’ (Supplemental file) with MS peptide tolerance of 5 ppm and fragment ion tolerance of 30 mmu. Tryptic peptides were accepted with one missed cleavage, and methionine oxidation was set as a variable modification. Target decoy was used with the strict FDR threshold of 1% and exported into Microsoft Excel (Microsoft, Redmond, WA, USA). The mass spectrometry proteomics data have been deposited to the ProteomeXchange Consortium via the PRIDE [37] partner repository with the dataset identifier P×D033687.

### Statistical analysis

Rarefaction curves, heatmaps, principal coordinates analyses (PCoA), Canonical Correspondence Analysis (CCA), and Wilcoxon rank-sum statistical tests were performed with R (R Foundation for Statistical Computing, Vienna, Austria) using the packages Vegan [38] and ade4 [39]. For PCoA analyses, data were first normalized using ANCOMBC and then Euclidean distances were calculated. Due to the small sample size, non-corrected *p*-values and species with >0.1% abundance were used when multiple comparisons were performed.

### Ethical considerations

The study was approved by the Regional Ethical Committee in Gothenburg, Sweden (Dnr 775–16) and the Swedish Data Inspection Board. Written informed consent was obtained from all patients.

## Results

### Clinical characteristics of study participants

Five patients with OLP and four controls had a systemic or extraoral disease. Only three subjects from the control group used regular medications (Table 1). One OLP patient and two controls used nasal glucocorticoids (fluticasone propionate and budesonide) to treat asthma and allergic asthma symptoms as needed. Although overall dental health was not examined in detail, no participant suffered from severe caries or periodontal disease in advanced stages or used removable dental prostheses.

**Table 1.** Demographics and clinical characteristics of the study population (N = 12).

Participant	Gender	Age (yr)	Diseases	Medications
OLP patient				
1	F	71	Atopic dermatitis; vitiligo	None
2	M	62	Asthma	Salmeterol and fluticasone propionate (prn)
3	M	60	None	None
4	F	59	Hypertension; breast cancer	None
5	F	56	Nephrolithiasis	None
6	F	38	Endometriosis; depression	None
Control				
1	F	66	None	None
2	M	64	None	None
3	M	62	Asthma; depression	Budesonide (prn); citalopram
4	F	57	Allergic asthma	Budesonide and albuterol (prn)
5	F	56	Hypertension; backpain	Candesartan; zopiclone; and NSAIDs
6	F	38	Depression	Citalopram; mirtazapine

OLP, oral lichen planus; prn, as needed.

### Metataxonomic profiling

Biological replicates from patients with OLP and controls yielded similar results in bacterial composition (Supplementary Figure S1). Thus, means from these two samples were calculated and used for each subject for statistical comparisons.

**Figure 1.**
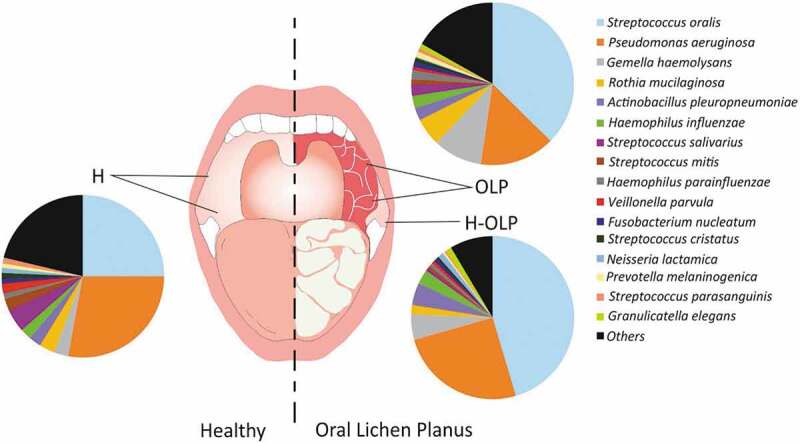
Microbiota associated with the buccal mucosa in healthy individuals and patients with Oral Lichen Planus (OLP). Sampling points are represented with lines, and the proportions of the bacterial species corresponding to each sample type are plotted in pie charts. Bacteria below 1% proportion were included in the ‘others’ category. H, healthy controls; OLP, affected sites; H-OLP; unaffected sites.

After quality filtering, on average, 7.5 × 10^4^ ±1.2 × 10^4^ reads were obtained per sample (OLP, 7.5 × 10^4^; H-OLP, 8.8 × 10^4^; and H, 7.6 × 10^4^) and annotated using the DADA2 program to the most similar ASVs at the different taxonomic levels. The most abundant species in the three sample types were *Streptococcus oralis* (23–47%) and *Pseudomonas aeruginosa* (10–23%), which together accounted for up to 70% of the total population (Figure 1). The remaining bacterial community was formed by 383 species, of which *Gemella haemolysans*, *Rothia mucilaginosa*, *Actinobacillus pleuropneumoniae, Haemophilus influenza*, *Streptococcus salivarius, Streptococcus mitis, Haemophilus parainfluenzae*, and *Veillonella parvula* presented the highest percentages (ranging from 2% to 9%) (Figure 1).

**Figure 2.**
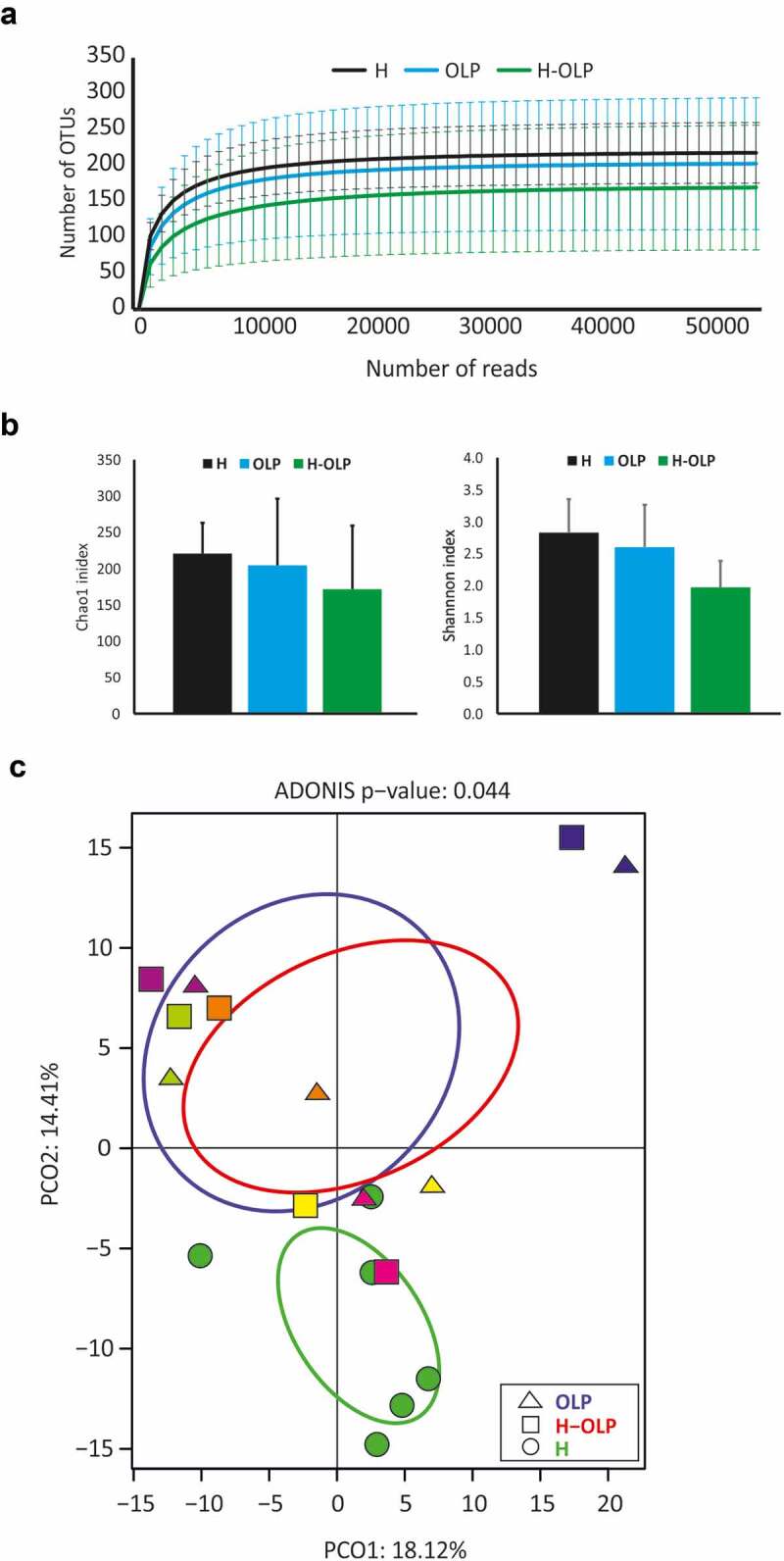
Differences in bacterial diversity and composition between healthy and OLP-associated oral samples. A, the number of amplicon sequence variants (ASVs) detected every 1000 reads plotted in rarefaction curves. B, Bar-graphs represent the richness index (Chao1) calculated for each sample type and the diversity index (Shannon) and their corresponding standard deviations. C, a Principal Coordinates Analysis (PCoA) plot based on the relative abundance of species-level ASVs. H, OLP, and H-OLP were differentiated using different shapes. Colours are used to associate affected (OLP) and unaffected (H-OLP) regions from the same patient.

Several approaches were used to compare the bacterial communities sampled from the different groups (OLP, H-OLP, and H) (Figure 2). Firstly, rarefaction curves were calculated in the three sample groups. The results showed asymptotic curves, indicating that most bacterial diversity was covered at the analysed sequencing depth. When the mean curves were compared (Figure 2A), the control group (H) showed a higher estimated number of species than patients with OLP (OLP and H-OLP), but the difference was not statistically significant. A non-significant lower number of species was also found in non-affected oral mucosa of patients with OLP (H-OLP) compared to affected sites (OLP) according to the rarefaction curves (Figure 2A), which was also confirmed by richness and diversity indices (Figure 2B). Samples were also plotted in a PCoA to show the differences in bacterial composition between samples (Figure 2C). Although there was some variability among individuals, sample clustering of the same group was observed, and the secondary component of the analysis separated OLP and H-OLP individuals from the controls. This suggested that microbial communities differed between patients with OLP and healthy individuals, and therefore more detailed analyses were performed to identify organisms potentially associated with OLP and health.

**Figure 3.**
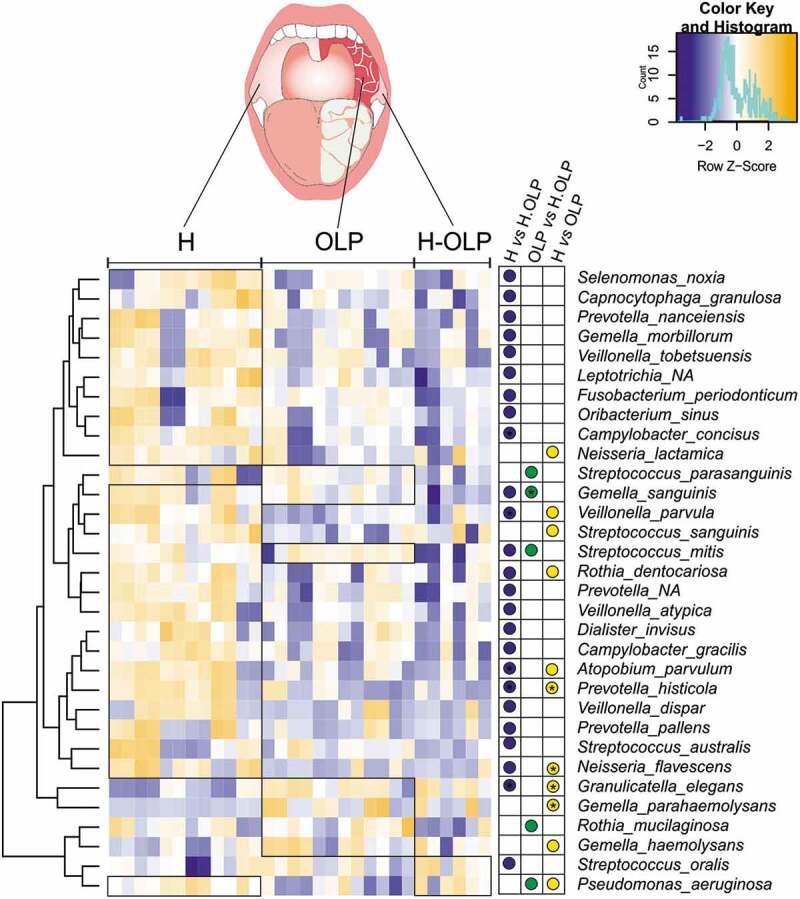
Heatmap showing differentially abundant genera of the buccal mucosal microbiota between healthy controls and patients with OLP. Both biological replicates were included for all samples; healthy controls (H), affected (OLP), and unaffected (H-OLP). The relative abundance of each species was normalized by ANCOM-BC, and their location clustered by their occurrence pattern in the three groups. Coloured circles indicate statistically significant differences between groups. * was used to highlight statistical differences using adjusted *p*-values.

A compositional analysis by ANCOM-BC revealed many species over-represented in samples from healthy individuals (Figure 3), including *P. aeruginosa*, four species of *Veillonella*, four species of *Prevotella*, three species of *Streptococcus* and two species of *Neisseria*. In contrast, a few species were over-represented in diseased sites, including *Granulicatella elegans*, *Streptococcus parasanguinis*, *Gemella haemolysans* and *Gemella parahaemolysans*. *G. haemolysans* was 3.4 times over-represented in patients with OLP compared to the controls, and its abundance reached almost 10% in the communities associated with OLP lesions.

Except for *Streptococcus oralis*, which appeared at higher levels in H-OLP, bacterial communities did not significantly differ between affected and non-affected sites of patients with OLP, suggesting that H-OLP sites were already affected by an equivalent bacterial dysbiosis and opening the possibility that bacteria uniquely associated with patients with OLP could be used as disease biomarkers.

### Metaproteomic profiling

To identify putative protein biomarkers in OLP lesions, we used mass spectrometry to detect the bacterial proteins expressed in our samples and compare their presence between affected and non-affected oral mucosa of patients with OLP. Consequently, peptides were annotated using a protein database based on the Human Oral Microbial Database (Supplementary Table S1), confirming the presence of bacteria identified by 16S rRNA sequencing. Most peptides corresponded to basic metabolic functions such as the 30S and 50S ribosomal subunits, DNA/RNA metabolism, or ABC transporters. Other peptides were annotated as hypothetical proteins, and their annotation could not be refined. Several peptides were putatively annotated as belonging to *G. haemolysans*, previously detected by our 16S rRNA analysis as a potential OLP biomarker. Also, many peptidases were found in the proteome data, potentially having a virulence function (Supplementary Table S2). Interestingly, some bacteria associated with the OLP group were identified in the metaproteome that were not amplified by the 16S rRNA gene sequencing approach, including the pathogen *Mycoplasma fermentans*.

## Discussion

Diverse factors, including genetic predisposition, immunological disturbances, infection, mechanical trauma, and stress, contribute to the etiopathogenesis of OLP. All these factors may result in microbial dysbiosis and alterations of metabolites leading to immune dysregulation and loss of integrity of the oral mucosa [40]. Previous studies have shown significant changes in the oral microbiome of patients with OLP [25–45]. Furthermore, a recent study reported reduced oral microbiota diversity and increased mucosal inflammation correlated with disease severity [46]. Also, histopathological features of OLP, such as atrophy, hyperkeratosis, acanthosis, and liquefaction of the basal layer, suggest barrier dysfunction [40]. In the present pilot study, we analysed the metataxonomic and metaproteomic profiles of the oral microbiome of patients with OLP and healthy individuals since a microbial signature for OLP is currently lacking. Several bacterial species, peptidases, and other proteins with inflammatory and virulence potential were identified in OLP patients.

Although evidence suggests that the oral microbiome of patients with OLP is significantly different compared to healthy individuals, the relatively few studies that have been published on this topic show divergent results. This could be attributed to numerous reasons, including the sample size, study design (cross-sectional vs longitudinal; lack of control groups), sampling method (saliva, oral rinse/mouthwash, swabs, and tissue biopsy, among others), and other methodological variables related to sample processing and data analysis. These variations also hinder the comparison of the results. Notably, most studies on OLP have used saliva samples to identify bacteria. Although saliva could be a reliable and practical source in the search for disease biomarkers as it captures the microbiota from all ecological niches of the oral cavity, it has been demonstrated that local or tissue samples (swabs and biopsies) are preferred in the investigation of potential etiopathogenic microorganisms in oral mucosal and non-mucosal diseases [45–49]. To date, only seven studies, including this, have collected tissue samples, i.e. biopsies, buccal swabs, or cytobrush, to analyse the oral microbiome of patients with OLP [26–28,43,45,50].

Previous studies have reported the differences in the microbial composition of OLP patients and healthy individuals using different approaches such as DNA-DNA hybridisation, PCR-denaturing gel electrophoresis or 16S rRNA gene sequencing [25,29,44]. Some of these focused on saliva, but most used oral swabs. In general, *Fusobacterium* and *Capnocytophaga* were associated with OLP lesions, whereas *Streptococcus* species were more associated with the oral mucosa of healthy controls. In agreement with this, in our study, several *Streptococcus* species were found to be more abundant in healthy individuals. Contrarily, *S. parasanguinis, Fusobacterium periodonticum* and *Capnocytophaga granulosa* were associated with OLP lesions compared to non-affected sites. Finally, and in agreement with previous reports using oral swabs [28,51] and saliva samples [45], we found a significant increase of *Gemella* in OLP patients both by Illumina sequencing and metaproteomics. The potential role of this bacterium in the development of OLP should be further investigated, given the recent data linking *G. haemolysans* with different infectious and inflammatory conditions ranging from allergies [31] to endocarditis, meningitis [52], and lactational mastitis [53].

Proteomic analysis using mass spectrometry has been performed previously in saliva samples of patients with OLP [54–57] but never through bacterial swabs as in the current study. Since *Veillonella tobetsuensis, V. atypica, V. dispar* and *V. parvula* were significantly reduced in patients with OLP, we hypothesised that the low proportion of this bacterium could be related to the development of the disease. This could represent an interesting biomarker, as *Veillonella* species use lactate as a carbon source, suggesting that the oral mucosa of patients with OLP could have reduced lactic acid levels. Furthermore, significantly higher proportions of other acidogenic and acidophilic bacteria, such as *Lactobacillus*, *Atopobium*, or *Scardovia* [58], were also found in healthy individuals. It should be noted that lactic acid not only results from bacterial metabolism but can also be overproduced by human lactate dehydrogenase activity in different mucosal tissues under stress conditions [59]. Therefore, lactate levels should be further analysed in OLP patients, as well as its potential use as a disease biomarker. In summary, although there is a high heterogeneity among these studies, it seems that an acidogenic and acidophilic community –as suggested by higher levels of lactate producers or users like *Veillonella, Lactobacillus*, or *Atopobium*– prevails in the oral mucosa of healthy individuals.

Metaproteomic profiling of patients with OLP (affected and non-affected sites) and controls showed peptides corresponding to basic metabolic functions such as the 30S and 50S ribosomal subunits, DNA/RNA metabolism, or ABC transporters. This is probably due to the dynamic range of the peptide concentrations that had been detected, implying that most hits correspond to housekeeping and highly expressed proteins. Remarkably, peptides belonging to *G. haemolysans*, a putative OLP biomarker, were detected. Among these, several peptidases were found, and two of them, one corresponding to an endopeptidase La and the other to an insulinase family protein, were identified in OLP lesions. Similarly, a Sapep family Mn(2+)-dependent dipeptidase and an M17 family metallopeptidase were detected in OLP lesions.

Among the detected proteins, an LCP family protein belonging to *G. haemolysans* is an evident virulence factor of several bacterial species, including *Streptococcus suis*, *Staphylococcus aureus*, and *Mycobacterium tuberculosis* [60]. Of note, several peptide hits corresponded to potential pathogens that the 16S rRNA sequencing data had not detected, perhaps because the wide-range primers extensively used for this kind of analysis do not properly amplify some bacteria with skewed genomic compositions like *Mycoplasma fermentans* [61]. The potential pathogenic role of this species in OLP should be explored, as it is considered a parasite of mucosal membranes, can be located intracellularly, and has been involved in several conditions, including chronic infections [62]. In addition, peptidases are accepted as common virulence factors used by bacteria to degrade host tissues. Thus, we also recommend future studies to evaluate the potential role of these peptidases in patients with OLP. It must also be considered that the short length of the peptides, the low coverage, and the database used might influence the annotation of peptides. Hence, future investigations with higher coverage and statistical power should confirm these results. Finally, it should be highlighted that, in this study, we did not implement protocols involving quantitative proteomic approaches. Mass spectrometry is not quantitative per se, so protocols involving label-free or labelling approaches should be applied to reach solid conclusions on differences in abundance. Another limitation of our study is the lack of a negative control during sampling and DNA extraction. Although we did include a negative control in the sequencing plaque, it is possible that *P. aeruginosa* is not part of the microbiota associated with the samples studied, considering that this bacterium is commonly regarded as a potential contaminant in oral samples. Finally, although an overall oral examination was performed in all participants, we cannot exclude the presence of interproximal caries lesions on contacting approximal surfaces or early-stage periodontal disease, which could influence the oral microbial composition.

In conclusion, considering the limitations of this study and the small sample size due to strict inclusion criteria, our findings suggest that several bacterial species could be associated with OLP. However, further longitudinal, properly controlled studies with larger cohorts, analysing tissue samples, are needed to understand the role of the oral microbiome in the pathogenesis of this condition and determine whether the presented microorganisms are valid disease biomarkers for OLP.

## Supplementary Material

Supplemental Material

## References

[1] Gonzalez-Moles MA, Warnakulasuriya S, Gonzalez-Ruiz I, et al. Worldwide prevalence of oral lichen planus: a systematic review and meta-analysis. Oral Dis. 2021;27(4):813–10. DOI:10.1111/odi.13323 32144836

[2] Li C, Tang X, Zheng X, et al. Global Prevalence and Incidence Estimates of Oral Lichen Planus: a Systematic Review and Meta-analysis. JAMA Dermatol. 2020;156(2):172–181. DOI:10.1001/jamadermatol.2019.3797 31895418 PMC6990670

[3] Robledo-Sierra J, Mattsson U, Jontell M. Use of systemic medication in patients with oral lichen planus - a possible association with hypothyroidism. Oral Dis. 2013;19(3):313–319. 22943671 10.1111/odi.12009

[4] Aghbari SMH, Abushouk AI, Attia A, et al. Malignant transformation of oral lichen planus and oral lichenoid lesions: a meta-analysis of 20095 patient data. Oral Oncol. 2017;68:92–102. 28438300 10.1016/j.oraloncology.2017.03.012

[5] Gonzalez-Moles MA, Ruiz-Avila I, Gonzalez-Ruiz L, et al. Malignant transformation risk of oral lichen planus: a systematic review and comprehensive meta-analysis. Oral Oncol. 2019;96:121–130. 31422203 10.1016/j.oraloncology.2019.07.012

[6] El-Howati A, Thornhill MH, Colley HE, et al. Immune mechanisms in oral lichen planus. Oral Dis. 2022. DOI:10.1111/odi.14142 35092132

[7] Carrozzo M, Porter S, Mercadante V, et al. Oral lichen planus: a disease or a spectrum of tissue reactions? Types, causes, diagnostic algorhythms, prognosis, management strategies. Periodontol 2000. 2019;80(1):105–125. 31090143 10.1111/prd.12260

[8] Galimanas V, Hall MW, Singh N, et al. Bacterial community composition of chronic periodontitis and novel oral sampling sites for detecting disease indicators. Microbiome. 2014;2(1):32. DOI:10.1186/2049-2618-2-32 25225610 PMC4164120

[9] Sartor RB, Wu GD. Roles for Intestinal Bacteria, Viruses, and Fungi in Pathogenesis of Inflammatory Bowel Diseases and Therapeutic Approaches. Gastroenterology. 2017;152(2):327–39 e4. 27769810 10.1053/j.gastro.2016.10.012 PMC5511756

[10] Holmstrup P, Schiotz AW, Westergaard J. Effect of dental plaque control on gingival lichen planus. Oral Surg Oral Med Oral Pathol. 1990;69(5):585–590. 2333211 10.1016/0030-4220(90)90241-j

[11] Salgado DS, Jeremias F, Capela MV, et al. Plaque control improves the painful symptoms of oral lichen planus gingival lesions. A short-term study. J Oral Pathol Med. 2013;42(10):728–732. 23721580 10.1111/jop.12093

[12] Backman K, Jontell M. Microbial-associated oral lichenoid reactions. Oral Dis. 2007;13(4):402–406. 17577327 10.1111/j.1601-0825.2006.01312.x

[13] Carbone M, Conrotto D, Carrozzo M, et al. Topical corticosteroids in association with miconazole and chlorhexidine in the long-term management of atrophic-erosive oral lichen planus: a placebo-controlled and comparative study between clobetasol and fluocinonide. Oral Dis. 1999;5(1):44–49. 10218041 10.1111/j.1601-0825.1999.tb00063.x

[14] Bankvall M, Sjoberg F, Gale G, et al. The oral microbiota of patients with recurrent aphthous stomatitis. J Oral Microbiol. 2014;6(1):25739. 25626771 10.3402/jom.v6.25739 PMC4221501

[15] Hijazi K, Lowe T, Meharg C, et al. Mucosal microbiome in patients with recurrent aphthous stomatitis. J Dent Res. 2015;94(3_suppl):87S–94S. 25540188 10.1177/0022034514565458 PMC4541092

[16] Dafar A, Bankvall M, Cevik-Aras H, et al. Lingual microbiota profiles of patients with geographic tongue. J Oral Microbiol. 2017;9(1):1355206. 28839519 10.1080/20002297.2017.1355206 PMC5560410

[17] Alam J, Lee A, Lee J, et al. Dysbiotic oral microbiota and infected salivary glands in Sjögren’s syndrome. PLoS ONE. 2020;15(3):e0230667. DOI:10.1371/journal.pone.0230667 32208441 PMC7092996

[18] Zhang X, Zhang D, Jia H, et al. The oral and gut microbiomes are perturbed in rheumatoid arthritis and partly normalized after treatment. Nat Med. 2015;21(8):895–905. DOI:10.1038/nm.3914 26214836

[19] Li BZ, Zhou HY, Guo B, et al. Dysbiosis of oral microbiota is associated with systemic lupus erythematosus. Arch Oral Biol. 2020;113:104708. 32203722 10.1016/j.archoralbio.2020.104708

[20] Lee BM, Park JW, Jo JH, et al. Comparative analysis of the oral microbiome of burning mouth syndrome patients. J Oral Microbiol. 2022;14(1):2052632. 35341209 10.1080/20002297.2022.2052632 PMC8942548

[21] Lodi G, Pellicano R, Carrozzo M. Hepatitis C virus infection and lichen planus: a systematic review with meta-analysis. Oral Dis. 2010;16(7):601–612. 20412447 10.1111/j.1601-0825.2010.01670.x

[22] Petti S, Rabiei M, De Luca M, et al. The magnitude of the association between hepatitis C virus infection and oral lichen planus: meta-analysis and case control study. Odontology. 2011;99(2):168–178. 21505737 10.1007/s10266-011-0008-3

[23] Jontell M, Watts S, Wallstrom M, et al. Human papilloma virus in erosive oral lichen planus. J Oral Pathol Med. 1990;19(6):273–277. 2169530 10.1111/j.1600-0714.1990.tb00841.x

[24] Syrjanen S, Lodi G, von Bultzingslowen I, et al. Human papillomaviruses in oral carcinoma and oral potentially malignant disorders: a systematic review. Oral Dis. 2011;Suppl 17:58–72. 10.1111/j.1601-0825.2011.01792.x 21382139

[25] Bornstein MM, Hakimi B, Persson GR. Microbiological findings in subjects with asymptomatic oral lichen planus: a cross-sectional comparative study. J Periodontol. 2008;79(12):2347–2355. 19053926 10.1902/jop.2008.080303

[26] Choi YS, Kim Y, Yoon HJ, et al. The presence of bacteria within tissue provides insights into the pathogenesis of oral lichen planus. Sci Rep. 2016;6(1):29186. DOI:10.1038/srep29186 27383402 PMC4935860

[27] He Y, Gong D, Shi C, et al. Dysbiosis of oral buccal mucosa microbiota in patients with oral lichen planus. Oral Dis. 2017;23(5):674–682. 28199766 10.1111/odi.12657

[28] Kragelund C, Keller MK. The oral microbiome in oral lichen planus during a 1-year randomized clinical trial. Oral Dis. 2019;25(1):327–338. 30144242 10.1111/odi.12961

[29] Wang K, Lu W, Tu Q, et al. Preliminary analysis of salivary microbiome and their potential roles in oral lichen planus. Sci Rep. 2016;6(1):22943. DOI:10.1038/srep22943 26961389 PMC4785528

[30] Kramer IR, Lucas RB, Pindborg JJ, et al. Definition of leukoplakia and related lesions: an aid to studies on oral precancer. Oral Surg Oral Med Oral Pathol. 1978;46:518–539. 280847

[31] Dzidic M, Collado MC, Abrahamsson T, et al. Oral microbiome development during childhood: an ecological succession influenced by postnatal factors and associated with tooth decay. Isme J. 2018;12(9):2292–2306. DOI:10.1038/s41396-018-0204-z 29899505 PMC6092374

[32] Hernandez M, Planells P, Martinez E, et al. Microbiology of molar–incisor hypomineralization lesions. A pilot study. J Oral Microbiol. 2020;12(1):1766166. 32595912 10.1080/20002297.2020.1766166 PMC7301705

[33] Callahan BJ, McMurdie PJ, Rosen MJ, et al. DADA2: high-resolution sample inference from Illumina amplicon data. Nat Methods. 2016;13(7):581–583. 27214047 10.1038/nmeth.3869 PMC4927377

[34] Quast C, Pruesse E, Yilmaz P, et al. The SILVA ribosomal RNA gene database project: improved data processing and web-based tools. Nucleic Acids Res. 2012;41:D590–6. 23193283 10.1093/nar/gks1219 PMC3531112

[35] Lin H, Peddada SD. Analysis of compositions of microbiomes with bias correction. Nat Commun. 2020;11:3514. 32665548 10.1038/s41467-020-17041-7 PMC7360769

[36] Kondori N, Kurtovic A, Pineiro-Iglesias B, et al. Mass Spectrometry Proteotyping-Based Detection and Identification of Staphylococcus aureus, Escherichia coli, and Candida albicans in Blood. Front Cell Infect Microbiol. 2021;11:634215. 34381737 10.3389/fcimb.2021.634215 PMC8350517

[37] Perez-Riverol Y, Bai J, Bandla C, et al. The PRIDE database resources in 2022: a hub for mass spectrometry-based proteomics evidences. Nucleic Acids Res. 2022;50:D543–52. 34723319 10.1093/nar/gkab1038 PMC8728295

[38] Oksanen J, Blanchet F, Kindt R, et al. Vegan: community Ecology Package. R package, ver. 2015:20–10

[39] Dray S, Dufour A-B. The ade4 Package: implementing the Duality Diagram for Ecologists. J Stat Softw. 2007;22(4):1–20.

[40] Lin D, Yang L, Wen L, et al. Crosstalk between the oral microbiota, mucosal immunity, and the epithelial barrier regulates oral mucosal disease pathogenesis. Mucosal Immunol. 2021;14(6):1247–1258. 34040155 10.1038/s41385-021-00413-7

[41] Carvalho M, Cavalieri D, Do Nascimento S, et al. Cytokines Levels and Salivary Microbiome Play a Potential Role in Oral Lichen Planus Diagnosis. Sci Rep. 2019;9:18137. 31792433 10.1038/s41598-019-54615-y PMC6889227

[42] Deng S, Xu Y, Wang X, et al. Study on the Role of Salivary Flora and NF-kappaB Inflammatory Signal Pathway in Oral Lichen Planus. Inflammation. 2020;43:994–1008. 32016629 10.1007/s10753-020-01185-1

[43] Du GH, Wang YF, Chen JJ, et al. Potential association between Fusobacterium nucleatum enrichment on oral mucosal surface and oral lichen planus. Oral Dis. 2020;26:122–130. 31710746 10.1111/odi.13232

[44] Wang K, Miao T, Lu W, et al. Analysis of oral microbial community and Th17-associated cytokines in saliva of patients with oral lichen planus. Microbiol Immunol. 2015;59(3):105–113. DOI:10.1111/1348-0421.12232 25644086

[45] Wang X, Zhao Z, Tang N, et al. Microbial Community Analysis of Saliva and Biopsies in Patients with Oral Lichen Planus. Front Microbiol. 2020;11:629. 32435231 10.3389/fmicb.2020.00629 PMC7219021

[46] Hijazi K, Morrison RW, Mukhopadhya I, et al. Oral bacterial diversity is inversely correlated with mucosal inflammation. Oral Dis. 2020;26(7):1566–1575. DOI:10.1111/odi.13420 32419230

[47] Al-Tarawneh SK, Border MB, Dibble CF, et al. Defining salivary biomarkers using mass spectrometry-based proteomics: a systematic review. OMICS. 2011;15:353–361. 21568728 10.1089/omi.2010.0134 PMC3125555

[48] Simon-Soro A, Tomas I, Cabrera-Rubio R, et al. Microbial geography of the oral cavity. J Dent Res. 2013;92(7):616–621. 23674263 10.1177/0022034513488119

[49] Varoni EM, Bavarian R, Robledo-Sierra J, et al. World Workshop on Oral Medicine VII: targeting the microbiome for oral medicine specialists—part 1. A methodological guide. Oral Dis. 2019;25(Suppl S1):12–27. DOI:10.1111/odi.13063 31140702

[50] Baek K, Lee J, Lee A, et al. Characterization of intratissue bacterial communities and isolation of Escherichia coli from oral lichen planus lesions. Sci Rep. 2020;10(1):3495. DOI:10.1038/s41598-020-60449-w 32103089 PMC7044275

[51] Yu FY, Wang QQ, Li M, et al. Dysbiosis of saliva microbiome in patients with oral lichen planus. BMC Microbiol. 2020;20(1):75. DOI:10.1186/s12866-020-01733-7 32245419 PMC7118920

[52] Garcia Lopez E, Martin-Galiano AJ. The Versatility of Opportunistic Infections Caused by Gemella Isolates is Supported by the Carriage of Virulence Factors from Multiple Origins. Front Microbiol. 2020;11:524. 32296407 10.3389/fmicb.2020.00524 PMC7136413

[53] Boix-Amoros A, Hernandez-Aguilar MT, Artacho A, et al. Human milk microbiota in sub-acute lactational mastitis induces inflammation and undergoes changes in composition, diversity and load. Sci Rep. 2020;10(1):18521. 33116172 10.1038/s41598-020-74719-0 PMC7595153

[54] Chaiyarit P, Taweechaisupapong S, Jaresitthikunchai J, et al. Comparative evaluation of 5–15-kDa salivary proteins from patients with different oral diseases by MALDI-TOF/TOF mass spectrometry. Clin Oral Investig. 2015;19(3):729–737. 10.1007/s00784-014-1293-3 25078551

[55] Souza MM, Florezi GP, Nico M, et al. Salivary proteomics in lichen planus: a relationship with pathogenesis? Oral Dis. 2018;24(5):784–792. 29383810 10.1111/odi.12837

[56] Talungchit S, Buajeeb W, Lerdtripop C, et al. Putative salivary protein biomarkers for the diagnosis of oral lichen planus: a case-control study. BMC Oral Health. 2018;18:42. 29534707 10.1186/s12903-018-0504-8 PMC5851270

[57] Yang LL, Liu XQ, Liu W, et al. Comparative analysis of whole saliva proteomes for the screening of biomarkers for oral lichen planus. Inflamm Res. 2006;55(10):405–407. 16850138 10.1007/s00011-006-5145-8

[58] Simon-Soro A, Mira A. Solving the etiology of dental caries. Trends Microbiol. 2015;23(2):76–82. 25435135 10.1016/j.tim.2014.10.010

[59] Tan TQ, Mason EJ, Wald ER, et al. Clinical characteristics of children with complicated pneumonia caused by Streptococcus pneumoniae. Pediatrics. 2002;110:1–6. 12093940 10.1542/peds.110.1.1

[60] Li F, Zhai D, Wu Z, et al. Impairment of the Cell Wall Ligase, LytR-CpsA-Psr Protein (LcpC), in Methicillin Resistant Staphylococcus aureus Reduces Its Resistance to Antibiotics and Infection in a Mouse Model of Sepsis. Front Microbiol. 2020;11:557. 32425893 10.3389/fmicb.2020.00557 PMC7212477

[61] Gonzalez JM, Portillo MC, Belda-Ferre P, et al. Amplification by PCR artificially reduces the proportion of the rare biosphere in microbial communities. PLoS ONE. 2012;7(1):e29973. 22253843 10.1371/journal.pone.0029973 PMC3256211

[62] Rosengarten R, Citti C, Glew M, et al. Host-pathogen interactions in mycoplasma pathogenesis: virulence and survival strategies of minimalist prokaryotes. Int J Med Microbiol. 2000;290(1):15–25. DOI:10.1016/S1438-42210080099-5 11043978

