# Supplementary material for: Metataxonomic and metaproteomic profiling of the oral microbiome in oral lichen planus - a pilot study
Source: J Oral Microbiol. 2022 Dec 28;15(1):2161726. doi: 10.1080/20002297.2022.2161726 (PMC9809343; doi:10.1080/20002297.2022.2161726)
Supplement: Supplemental Material [file ZJOM_A_2161726_SM1517.zip › Supplementary Table 1.docx]

| **Feature** | **H** | **OLP** | **H-OLP** |
| --- | --- | --- | --- |
| *Atopobium parvulum* | 0.18 | 0.04 | 0.02 |
| *Campylobacter concisus* | 0.23 | 0.08 | 0.03 |
| *Campylobacter gracilis* | 0.11 | 0.06 | 0.04 |
| *Capnocytophaga granulosa* | 0.11 | 0.05 | 0.03 |
| *Dialister invisus* | 0.13 | 0.07 | 0.02 |
| *Fusobacterium periodonticum* | 0.37 | 0.003 | 0.05 |
| *Gemella haemolysans* | 2.87 | 9.77 | 5.06 |
| *Gemella morbillorum* | 0.17 | 0.04 | 0.03 |
| *Gemella parahaemolysans* | 0.00 | 0.99 | 0.43 |
| *Gemella sanguinis* | 0.34 | 0.19 | 0.06 |
| *Granulicatella elegans* | 0.06 | 0.91 | 1.17 |
| *Leptotrichia NA* | 0.42 | 0.14 | 0.07 |
| *Neisseria flavescens* | 0.34 | 0.02 | 0.02 |
| *Neisseria lactamica* | 1.02 | 0.28 | 1.14 |
| *Oribacterium sinus* | 0.36 | 0.02 | 0.03 |
| *Prevotella histicola* | 0.19 | 0.03 | 0.01 |
| *Prevotella NA* | 0.68 | 0.44 | 0.13 |
| *Prevotella nanceiensis* | 0.12 | 0.02 | 0.01 |
| *Prevotella pallens* | 0.10 | 0.02 | 0.01 |
| *Pseudomonas aeruginosa* | 27.90 | 14.93 | 25.21 |
| *Rothia dentocariosa* | 1.26 | 0.42 | 0.32 |
| *Rothia mucilaginosa* | 3.07 | 5.56 | 1.84 |
| *Selenomonas noxia* | 0.13 | 0.04 | 0.02 |
| *Streptococcus australis* | 0.36 | 0.04 | 0.01 |
| *Streptococcus mitis* | 0.61 | 0.87 | 0.23 |
| *Streptococcus oralis* | 24.99 | 37.38 | 45.35 |
| *Streptococcus parasanguinis* | 1.09 | 0.74 | 0.35 |
| *Streptococcus sanguinis* | 0.43 | 0.24 | 0.33 |
| *Veillonella atypica* | 0.90 | 0.18 | 0.10 |
| *Veillonella dispar* | 0.12 | 0.16 | 0.05 |
| *Veillonella parvula* | 1.92 | 0.60 | 0.57 |
| *Veillonella tobetsuensis* | 0.14 | 0.05 | 0.03 |
|  |  |  |  |

**Supplementary Table 1.** Frequency of bacterial species differentially represented in the three studied groups. Only bacteria at >0.1% average frequency are shown for clarity.

OLP, oral lichen planus; H-OLP, healthy sites of OLP patients; H, healthy controls.
