# Supplementary figures and images for: Metataxonomic and metaproteomic profiling of the oral microbiome in oral lichen planus - a pilot study
Source: J Oral Microbiol. 2022 Dec 28;15(1):2161726. doi: 10.1080/20002297.2022.2161726 (PMC9809343; doi:10.1080/20002297.2022.2161726)

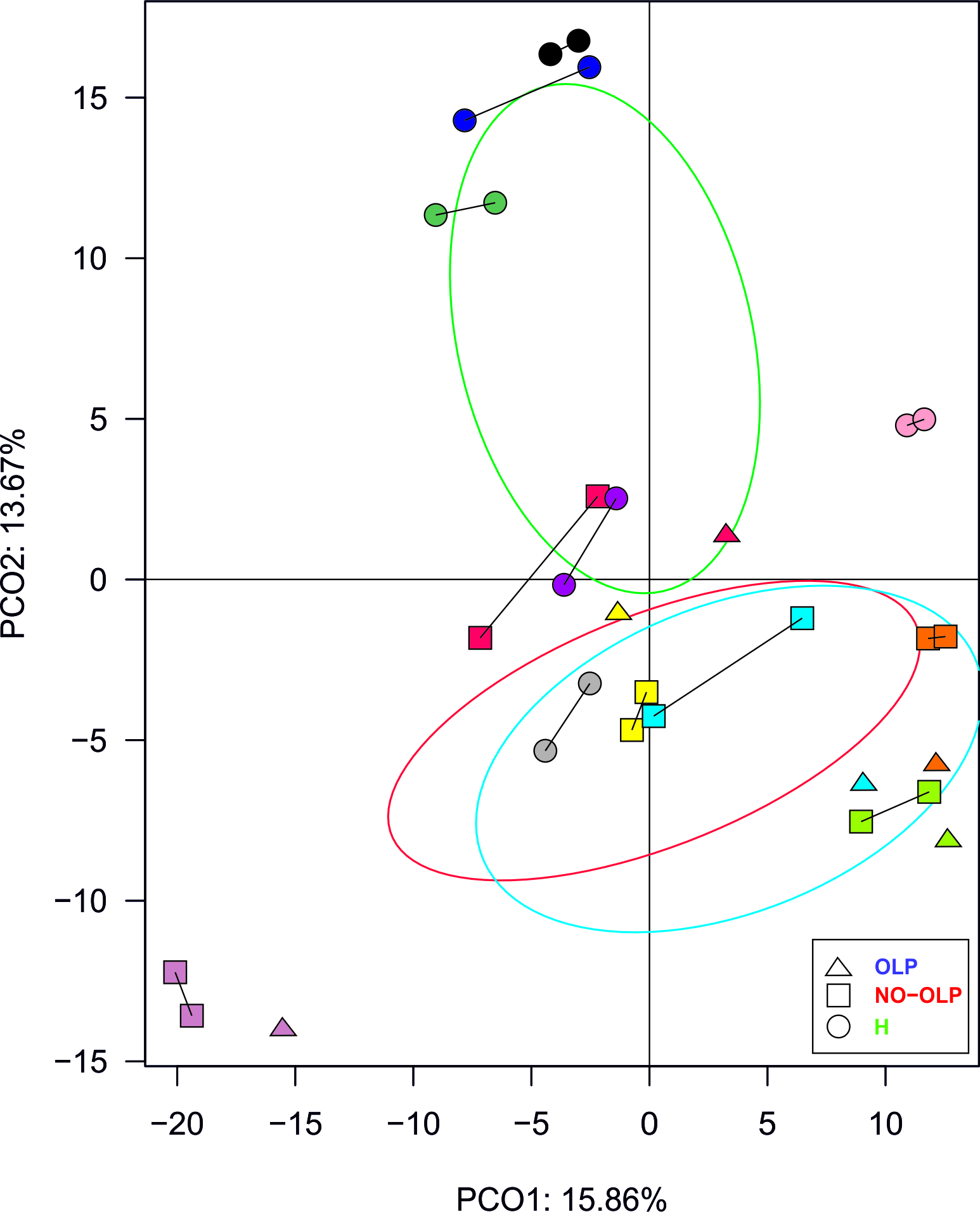

Supplement: Supplemental Material [file ZJOM_A_2161726_SM1517.zip › Supplementary Figure 1.jpg]
